# Comparison of the clinical and prognostic characteristics of patients with different pathological types in acromegaly

**DOI:** 10.3389/fendo.2025.1571598

**Published:** 2025-05-12

**Authors:** Liye Chong, Yuxing Lou, Xue Chen, Wenji Zhao, Wei Zhang, Ziwei Zhang, Fan Yang, Ping Li

**Affiliations:** ^1^ Department of Endocrinology, Endocrine and Metabolic Disease Medical Center, Nanjing Drum Tower Hospital, Affiliated Hospital of Medical School, Nanjing University, Nanjing, China; ^2^ Nanjing Drum Tower Hospital, Branch of National Clinical Research Center for Metabolic Diseases, Nanjing, China; ^3^ Department of Endocrinology, Endocrine and Metabolic Disease Medical Center, Nanjing Drum Tower Hospital Clinical College of Nanjing University of Chinese Medicine, Nanjing, China; ^4^ Department of Pathology, Drum Tower Hospital, Medical School of Nanjing University, Nanjing, China

**Keywords:** acromegaly, PIT1/SF1 tumor, GH/PRL positive tumor, clinical characteristics, prognosis

## Abstract

**Context:**

Acromegaly is caused by somatotroph tumors. Recently, the WHO recommended the use of transcription factors (TFs) together with pituitary hormones to accurately classify the subtypes.

**Objective:**

This study aims to evaluate differences in the clinical and prognostic characteristics of acromegaly patients with different pathological types.

**Methods:**

A retrospective study was conducted on 94 acromegaly patients who underwent surgical treatment. Patients were classified into two groups on the basis of TFs expression by IHC. PIT1 tumors were positive only for PIT1, and PIT1/SF1 tumors were positive for both PIT1 and SF1. Additionally, on the basis of the expression of GH and PRL by IHC, PIT1 tumors were further subdivided into GH positive tumors (those positive for only GH) and GH/PRL positive tumors (those positive for both GH and PRL). Differences in clinical and prognostic features among the pathological groups were evaluated.

**Results:**

PIT1/SF1 tumors represented 30.9% (n = 29) of the acromegaly patients in this cohort. PIT1/SF1 tumors had a higher baseline IGF-1 index (2.77 ± 0.73 vs. 2.39 ± 0.74, *P* = 0.024) than PIT1 tumors. Despite the higher proportion of postoperative GH < 1 μg/L, the biochemical remission rate of PIT1/SF1 tumors (30.8% vs. 27.6%, *P* = 0.812) was similar to that of PIT1 tumors. Compared with those with GH positive tumors, patients with GH/PRL positive tumors were younger at diagnosis (42.50 ± 13.36 vs. 49.05 ± 11.69, *P* = 0.046), and the proportion of male patients was higher (50.0% vs. 23.3%, *P* = 0.048). Furthermore, patients with GH/PRL positive tumors had a significantly higher postoperative GH level [7.30 (3.18–11.08) vs. 2.49 (1.57–6.84), *P* = 0.011] and IGF-1 index (1.82 ± 0.94 vs. 1.31 ± 0.63, *P* = 0.011) during follow-up. The biochemical remission rate in GH/PRL positive tumors was lower, but the difference was not statistically significant (18.2% vs. 37.2%, *P* = 0.159).

**Conclusion:**

PIT1/SF1 tumors represent approximately 30.0% of acromegaly patients. Despite higher baseline IGF-1 levels, the clinical and prognostic features of patients with PIT1/SF1 tumors are similar to those of patients with PIT1 tumors. GH/PRL positive tumors, characterized by their earlier age at diagnosis and male predominance, tend to exhibit a lower biochemical remission rate compared to GH positive tumors.

## Introduction

Acromegaly is a chronic systemic disease characterized by elevated growth hormone (GH) and insulin-like growth factor 1 (IGF-1) levels (1). Chronic excess GH and IGF-1 lead to multisystemic complications, including cardiovascular disease, osteoarthropathy, and metabolic disorders (2). More than 99% of acromegaly cases are attributed to pituitary somatotroph adenomas (3). According to the WHO classification of pituitary adenoma (PA), which is based primarily on the expression of pituitary hormones detected by immunohistochemistry (IHC), somatotroph adenomas are categorized as densely granulated somatotroph adenoma, sparsely granulated somatotroph adenoma, mammosomatotroph adenoma, or mixed somatotroph and lactotroph adenoma (4). With further studies on the pathogenesis and biological behavior of PAs, the proposal to change the nomenclature from PA to pituitary neuroendocrine tumor (PitNET) was adopted (5). Recently, the WHO updated the classification and recommended the use of pituitary transcription factors (TFs) and pituitary hormones to determine cell lineages for the subclassification of PitNETs (6). PIT1 (pituitary transcription factor 1), encoded by the POU1F1 gene, determines the differentiation of somatotrophs, lactotrophs, and thyrotrophs. SF1 (steroidogenic factor 1), encoded by the NR5A1 gene, regulates gonadotroph cell differentiation. TPIT (T-box pituitary transcription factor), encoded by the TBX19 gene, is responsible for the development of corticotroph cells.

Pituitary TFs play crucial roles in determining the differentiation of adenohypophyseal stem cells and accurately classifying the subtypes of PitNETs (6). With the routine application of TFs, studies have reported few acromegaly patients harboring atypical tumors that stain positive for both PIT1 and SF1 (PIT1/SF1 tumors) (7–9). Few studies on PIT1/SF1 tumors have been conducted and the clinical and prognostic characteristics of these tumors remain uncertain. Additionally, approximately 16–27% of acromegaly patients present elevated GH and prolactin (PRL) levels (10). The GH and PRL cosecreting tumors have garnered increased attention in recent years. However, the definition and prognosis of these tumors are still controversial.

To address these knowledge gaps, we retrospectively reviewed medical records and conducted a comprehensive comparative analysis between PIT1/SF1 and PIT1 tumors in a large cohort of operated acromegaly patients. Furthermore, according to the 2022 WHO classification of PitNETs, acromegaly patients harboring PIT1 tumors were further categorized on the basis of the expression of GH and PRL by IHC. In this study, we aimed to assess the differences in the demographic, imaging, pathological, metabolic, and prognostic features of acromegaly patients with different pathological types.

## Materials and methods

### Patients and inclusion criteria

We retrospectively reviewed the clinical data of acromegaly patients who underwent surgical treatment at Nanjing Drum Tower Hospital between January 2017 and May 2024. The diagnostic criteria for acromegaly were as follows (11): (1) characteristic clinical signs and symptoms related to excessive GH and IGF-1; (2) nadir GH > 1.0 μg/L during an oral glucose tolerance test (OGTT); and (3) IGF-1 levels above the upper limit of normal (ULN) adjusted for age. Patients who met the following criteria were included: (1) newly diagnosed with acromegaly who underwent surgery for somatotroph tumors; (2) sufficient preoperative and postoperative demographic, imaging, pathological, metabolic, hormonal, and prognostic data; and (3) regular follow-up at least three months after surgery. Patients who had undergone surgery, medical therapy (somatostatin receptor ligand, dopamine agonist, or GH receptor antagonist) or radiotherapy for tumors prior to hospital admission were excluded from the study.

### Endocrinological evaluation and biochemical remission after the operation

All patients underwent an OGTT after an overnight fast for the diagnosis of acromegaly and assessment of glucose metabolism status. Blood samples were simultaneously collected to measure plasma glucose, insulin, and serum GH levels at 0, 30, 60, 90, and 120 minutes after the oral administration of 75 g of glucose. The nadir GH level was defined as the lowest GH level measured during the OGTT. Abnormal glucose metabolism includes impaired fasting glucose, impaired glucose tolerance, and diabetes mellitus (12). The homeostasis model assessment (HOMA) was utilized to evaluate insulin resistance and pancreatic β-cell function (13, 14).

The samples were also submitted to the same laboratory for the measurement of biochemical parameters and hormone levels. Serum GH levels were determined with a two-site chemiluminescent immunometric assay (Immulite 2000, Siemens Healthcare Diagnostics Products Limited) with a detection range of 0.05–40 μg/L and an analytical sensitivity of 0.01 μg/L. Serum IGF-1 levels were measured with a solid-phase chemiluminescent immunometric assay (Immulite 2000, Siemens Healthcare Diagnostics Products Limited) with a detection range of up to 1000 ng/mL. To ensure that the IGF-1 levels were comparable between patients, the IGF-1 index was used to standardize the IGF-1 levels. IGF-1 index= IGF-1/ULN adjusted for age. In particular, the normal range of PRL varies by sex, being 2.1–17.7 μg/L for men, 2.8–29.2 μg/L for premenopausal and nonpregnant women, 9.7–208.5 μg/L for pregnant women, and 1.8–20.3 μg/L for postmenopausal women.

Postoperative GH was defined as random serum GH within one day after surgery. Biochemical remission was defined as a random serum GH < 1.0 μg/L with normalization of serum IGF-1 adjusted for age at three months after surgery (11).

### Imaging evaluation

To evaluate the imaging characteristics of tumors preoperatively and postoperatively, each patient underwent enhanced 3.0 T magnetic resonance imaging (MRI). Data on the maximum diameter of the tumor, compression of the optic nerve, invasion of the cavernous sinus, and internal carotid artery were obtained and recorded. Based on the maximum diameter, pituitary adenomas were categorized as microadenomas (< 1 cm) or macroadenomas (≥ 1 cm). The Knosp grade (grade 0–4) was determined from coronal sections of T1-weighted contrast-enhanced MRI images, and tumors were considered invasive if the Knosp grade was 3 or 4.

### Pathology

The resected tissue was immediately fixed in 10% buffered formalin and subsequently embedded in paraffin. Blocks were cut into 3–4 µm paraffin sections and then stained with hematoxylin and eosin. IHC staining of the sections was uniformly performed on an automated IHC system (Ultra 60, ZSGB-Bio Co., Ltd., CHN). Immunohistochemical analysis was conducted for cytokeratin, the proliferation marker Ki-67 and hormonal markers, including GH, PRL, thyroid-stimulating hormone (TSH), luteinizing hormone (LH), follicle-stimulating hormone (FSH) and adrenocorticotropic hormone (ACTH) for each tumor. Antibodies against PIT, SF1, and TPIT (all monoclonal, prediluted, ZSGB-Bio Co., Ltd., CHN) were used to detect TF expression in the tumor samples. The sections were reviewed by a board-certified neuropathologist who was blinded to the patients’ medical records.

On the basis of the expression of TFs by IHC, acromegaly patients were classified into two groups: PIT1 tumors (defined as a positive stain only for PIT1) and PIT1/SF1 tumors (defined as a positive stain for both PIT1 and SF1). Additionally, on the basis of GH and PRL expression by IHC, PIT1 tumors were further subdivided into GH positive tumors (those positive for only GH) and GH/PRL positive tumors (those positive for both GH and PRL). Specifically, tumors in which PRL staining was positive in more than 10% of the cells were classified as PRL positive (15). GH positive tumors, also known as somatotroph tumors, include densely granulated somatotroph tumors and sparsely granulated somatotroph tumors. Multiple types of PIT1 lineage PitNETs can manifest as acromegaly and stain positive for both GH and PRL by IHC, including mammosomatotroph tumors (MSTs), mixed somatotroph and lactotroph tumors (MSLTs), and acidophil stem cell tumors. Given that precise differentiation between these tumor subtypes relies on electron microscopy, they were grouped together into GH/PRL positive tumor group.

### Statistical analysis

Statistical analyses were conducted with SPSS (version 27.0, IBM, USA), and graphs were generated with GraphPad Prism (version 9.1.0.221, GraphPad, USA). Continuous variables are expressed as the mean ± standard deviation for normally distributed data or as median with interquartile range (IQR) for nonnormally distributed data. Student’s t test and the Mann–Whitney U test were performed for between-group comparisons. Categorical variables are presented as numbers and percentages and were compared by means of the chi-square test or Fisher’s exact test. A two-tailed *P* < 0.05 was considered to indicate statistically significant differences.

## Results

### Patient characteristics

In total, 109 acromegaly patients who underwent surgery were enrolled in the study, 12 patients were excluded because of insufficient medical records, and 3 patients were excluded due to a short follow-up (less than three months after surgery). Finally, 94 patients were included in the analysis. The characteristics of these patients at baseline are summarized in **Table 1**. The average age at diagnosis was 47.37 ± 12.25 years, with a median disease duration of 60 months. There were 64 female patients (68.1%), and the vast majority of patients (80.9%) harbored macroadenomas. At baseline, the median random GH level was 11.45 μg/L (IQR 7.05–29.23), and the nadir GH level during the OGTT was 9.34 μg/L (IQR 5.27–27.30). The mean IGF-1 index in these patients was 2.51 ± 0.75.

**Table 1:** General and clinical characteristics of acromegaly patients at baseline.

Varible	n = 94
Age at diagnosis (years)	47.37 ± 12.25
Female (n, %)	64 (68.1%)
BMI (kg/m^2^)	26.05 ± 2.70
Disease duration (months)	60.00 (24.00–120.00)
Macroadenoma (n, %)	76 (80.9%)
Baseline random GH (μg/L)	11.45 (7.05–29.23)
Baseline nadir GH (μg/L)	9.34 (5.27–27.30)
Baseline IGF-1 (ng/mL)	620.00 (514.50–775.00)
Baseline IGF-1 index	2.51 ± 0.75

BMI, body mass index; GH, growth hormone; IGF-1, insulin-like growth factor-1.

### Clinical characteristics and treatment outcomes of patients with PIT1 and PIT1/SF1 tumors

**Figure 1:**
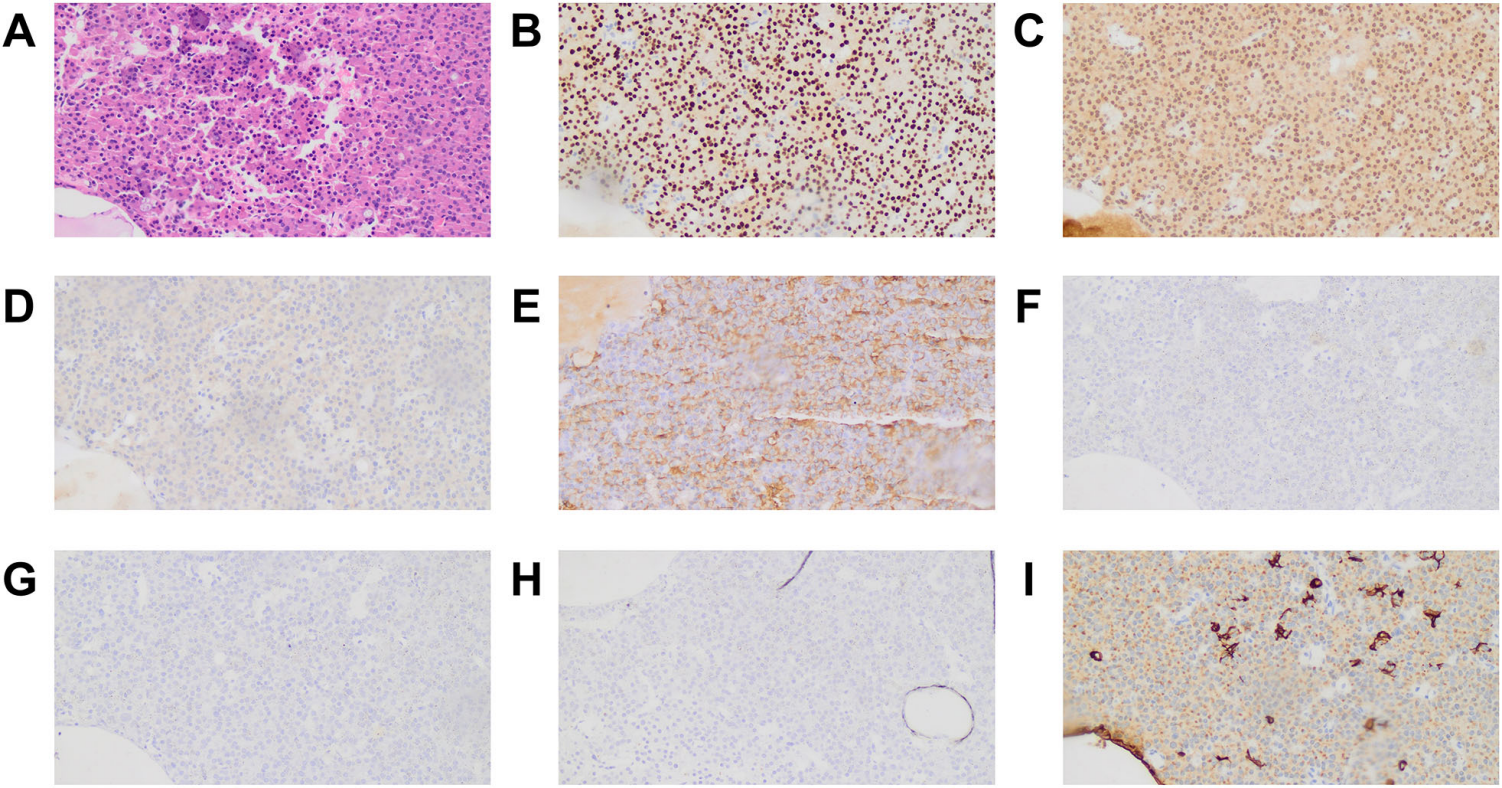
Histological appearance and immunostaining profile of a PIT1/SF1 tumor. The tumor was composed of a monomorphic cell population with abundant eosinophilic cytoplasm on hematoxylin and eosin stain **(A)**. Tumor cells exhibited intense nuclear reactivity for PIT1 **(B)** and SF1 **(C)** but were negative for TPIT **(D)**. Immunostaining for GH was obviously and diffusely positive **(E)**, whereas immunostaining for PRL **(F)**, FSH **(G)** and LH **(H)** was negative. Some tumor cells displayed a perinuclear positivity pattern on CK immunostaining, whereas others presented fibrous bodies in the cytoplasm **(I)**. All microphotographs were performed on magnification ×200.

All tumors were positive for PIT1 and negative for TPIT. Notably, 29 tumors stained positive for both PIT1 and SF1. Therefore, on the basis of the expression of TFs by IHC, patients were classified into PIT1 and PIT1/SF1 tumors. PIT1 tumors accounted for 69.1% (n = 65) of patients, whereas PIT1/SF1 tumors accounted for the other 30.9%. **Figure 1** shows the histological appearance and immunostaining profile of a representative PIT1/SF1 tumor.

As shown in **Table 2**, there were no significant differences in demographic characteristics, including age at diagnosis, sex distribution, BMI and disease duration, between the two groups. The PIT1 tumors did not differ from the PIT1/SF1 tumors in terms of tumor diameter or the proportions of macroadenomas and invasive tumors (Knosp grade 3–4). Since SF1 regulates gonadotroph cell differentiation, we assessed the expression of FSH and LH in these tumors. Only 2 of 29 tumors in the PIT/SF1 group stained positive for FSH or LH, while the expression of these hormones was absent in the PIT1 tumors. Pathological studies revealed that PIT1/SF1 tumors more frequently displayed a perinuclear cytokeratin pattern than PIT1 tumors (86.2% vs. 52.3%, *P* = 0.002), whereas few PIT1/SF1 tumors presented fibrous bodies in the cytoplasm. The glucose metabolism status and pancreatic β-cell function, as evaluated by HOMA-β, were similar in the two cohorts. Moreover, no differences in the incidence of abnormal glucose metabolism or hypertension were found between PIT1 and PIT1/SF1 tumors.

**Table 2:** Clinical characteristics and treatment outcomes of patients with PIT1 and PIT1/SF1 tumors.

Varible	PIT1 (n = 65)	PIT1/SF1 (n = 29)	*P* value
Demographics			
Age at diagnosis (years)	46.83 ± 12.57	48.59 ± 11.61	0.524
Female (n, %)	44 (67.7%)	20 (69.0%)	1.000
BMI (kg/m^2^)	25.97 ± 2.77	26.23 ± 2.58	0.671
Disease duration (months)	60.00 (18.00–120.00)	60.00 (24.00–120.00)	0.363
Imaging performance			
Maximum tumor diameter (cm)	1.70 (1.10–2.45)	1.70 (1.05–2.75)	0.755
Macroadenoma (n, %)	54 (83.1%)	22 (75.9%)	0.571
Optic nerve compression (n, %)	19 (29.2%)	10 (34.5%)	0.635
Knosp grade 3–4 (n, %)	22 (33.8%)	9 (31.0%)	0.818
Pathology			
Cytokeratin pattern			**0.002**
Perinuclear (n, %)	34 (52.3%)	25 (86.2%)	
Fibrous body (n, %)	31 (47.7%)	4 (13.8%)	
Ki-67 index > 3% (n, %)	8 (12.3%)	7 (24.1%)	0.221
Metabolism characteristic			
Abnormal glucose metabolism (n, %)	43 (66.2%)	20 (69.0%)	0.789
HbA1c (%)	6.20 (5.35–8.30)	6.20 (5.93–6.93)	0.504
FPG (mmol/L)	5.71 (5.01–7.86)	5.82 (5.32–7.09)	0.641
Fasting insulin (mIU/L)	15.76 (7.83–19.15)	15.43 (9.94–2.66)	0.336
HOMA-β	124.83 (55.46–212.96)	139.02 (79.58–190.31)	0.586
HOMA-IR	3.93 (2.37–5.96)	4.63 (2.53–6.01)	0.366
TyG index	8.90 ± 0.74	8.89 ± 0.50	0.956
Hypertension (n, %)	26 (40.0%)	13 (44.8%)	0.821
Preoperation			
Baseline random GH (μg/L)	10.20 (6.70–34.45)	12.60 (7.72–27.60)	0.552
Baseline nadir GH (μg/L)	8.48 (5.03–27.05)	11.10 (6.93–27.75)	0.348
Baseline IGF-1 (ng/mL)	593.00 (476.00–741.00)	675.00 (538.00–804.50)	0.094
Baseline IGF-1 index	2.39 ± 0.74	2.77 ± 0.73	**0.024**
Hypopituitarism (n, %)	14 (21.5%)	6 (20.7%)	1.000
Postoperation			
Postoperative GH (μg/L)	3.28 (1.77–8.90)	2.33 (0.87–5.15)	0.150
Postoperative GH < 1μg/L (n,%)	8 (12.3%)	10 (34.5%)	**0.021**
Follow-up			
Follow-up duration (months)	12.00 (6.00–33.00)	18.00 (6.00–56.00)	0.254
Random GH at follow-up (μg/L)	2.27 (0.53–4.72)	1.54 (0.58–3.30)	0.595
IGF-1 at follow-up (ng/mL)	293.00 (199.00–499.00)	281.00 (201.50–415.00)	0.703
IGF-1 index at follow-up	1.38 (0.92–1.93)	1.27 (0.88–1.77)	0.787
Biochemical remission (n, %)	20 (30.8%)	8 (27.6%)	0.812
Second operation (n, %)	4 (6.2%)	1 (3.4%)	1.000

PIT1, pituitary transcription factor 1; SF1, steroidogenic factor 1; BMI, body mass index; HbA1c, glycated hemoglobin A1c; FPG, fasting plasma glucose; HOMA-β, homeostasis model assessment for β cell function; HOMA-IR, homeostasis model assessment for insulin resistance; TyG, triglyceride-glucose; GH, growth hormone; IGF-1, insulin-like growth factor-1.

The bold values represent the P-values that are statistically significant.

Before surgery, although baseline random GH and nadir GH levels during the OGTT did not significantly differ among the groups, we observed that PIT1/SF1 tumors had a higher IGF-1 level [675.00 (538.00–804.50) vs. 593.00 (476.00–741.00), *P* = 0.094] and mean IGF-1 index (2.77 ± 0.73 vs. 2.39 ± 0.74, *P* = 0.024) than PIT1 tumors at baseline. After surgery, the serum GH levels were significantly decreased, and the proportion of postoperative GH < 1 μg/L among the PIT1/SF1 tumors was higher than that among the PIT1 tumors (34.5% vs. 12.3%, *P* = 0.021). However, the differences in the biochemical remission rates did not reach significance (30.8% vs. 27.6%, *P* = 0.812). More details regarding treatment outcomes are listed in **Table 2**.

### Clinical characteristics and treatment outcomes of patients with GH positive and GH/PRL positive tumors

**Figure 2:**
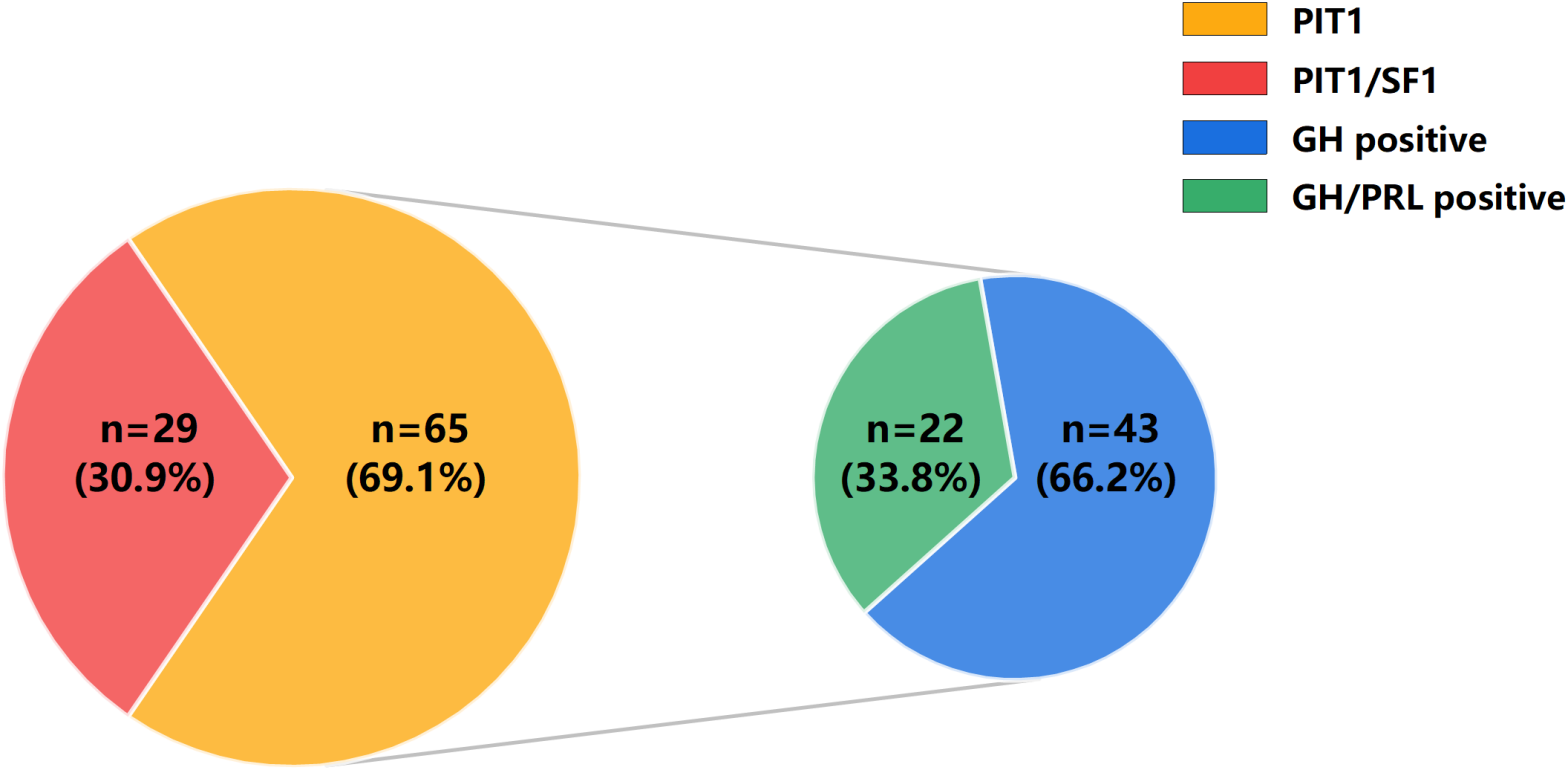
Distribution of acromegaly patients with different pathological types. On the basis of the expression of TFs by IHC, 94 acromegaly patients were classified into PIT1 tumors (n = 65) and PIT1/SF1 tumors (n = 29). Additionally, on the basis of the expression of GH and PRL by IHC, PIT1 tumors were further subdivided into GH positive tumors (n = 43) and GH/PRL positive tumors (n = 22).

PIT1 tumors were further subdivided into GH positive and GH/PRL positive tumors on the basis of the expression of GH and PRL by IHC. Forty-three patients (66.2%) had GH positive tumors, whereas 22 patients (33.8%) had GH/PRL positive tumors. **Figure 2** illustrates the patterns and incidences of these subgroups. Moreover, we further characterized the GH and PRL staining profiles of PIT1/SF1 tumors. Among 29 PIT1/SF1 tumors, 62.1% (n = 18) stained positive for only GH and 37.9% (n = 11) stained positive for both GH and PRL, which was similar to the staining profiles observed in the PIT1 tumors (62.1% vs. 66.2% *P* = 0.816).

The demographic, imaging, pathological, metabolic characteristics, and treatment outcomes of these patients are presented in **Table 3**. The age at diagnosis was lower in the GH/PRL positive tumors than in the GH positive tumors (42.50 ± 13.36 vs. 49.05 ± 11.69, *P* = 0.046). Moreover, the proportion of male patients in the GH/PRL positive tumors was higher than that in the GH positive tumors (50.0% vs. 23.3%, *P* = 0.048). In terms of imaging characteristics, no differences were found among the groups regarding tumor diameter or the prevalence of macroadenomas or invasive tumors. IHC staining of resected samples revealed that GH/PRL positive tumors more frequently exhibited a perinuclear cytokeratin pattern than GH positive tumors (86.4% vs. 34.9%, *P* < 0.001). With respect to metabolic comorbidities, we observed no significant difference in glucose metabolism parameters or the incidence of hypertension between the two groups.

**Table 3:** Clinical characteristics and treatment outcomes of patients with GH positive and GH/PRL positive tumors.

Variable	GH positive (n = 43)	GH/RPL positive (n = 22)	*P* value
Demographics			
Age at diagnosis (years)	49.05 ± 11.69	42.50 ± 13.36	**0.046**
Female (n, %)	33 (76.7%)	11 (50.0%)	**0.048**
BMI (kg/m^2^)	25.73 ± 2.63	26.44 ± 3.03	0.334
Disease duration (months)	60.00 (24.00–120.00)	48.00 (12.00–120.00)	0.802
Imaging performance			
Maximum tumor diameter (cm)	1.90 (1.10–2.60)	1.65 (0.99–2.33)	0.537
Macroadenoma (n, %)	37 (86.0%)	17 (77.3%)	0.487
Optic nerve compression (n, %)	15 (34.9%)	4 (18.2%)	0.249
Knosp grade 3–4 (n, %)	17 (39.5%)	5 (22.7%)	0.268
Pathology			
Cytokeratin pattern			**< 0.001**
Perinuclear (n, %)	15 (34.9%)	19 (86.4%)	
Fibrous body (n, %)	28 (65.1%)	3 (13.6%)	
Ki-67 index > 3% (n, %)	5 (11.6%)	3 (13.6%)	1.000
Metabolic characteristic			
Abnormal glucose metabolism (n, %)	29 (67.4%)	14 (63.6%)	0.759
HbA1c (%)	6.10 (5.30–8.20)	6.60 (5.38–8.00)	0.483
FPG (mmol/L)	5.56 (4.92–7.96)	6.02 (5.05–8.17)	0.462
Fasting insulin (mIU/L)	15.76 (6.99–19.20)	12.87 (7.58–19.18)	0.642
HOMA-β	128.99 (59.04–221.20)	111.49 (34.23–180.04)	0.438
HOMA-IR	3.93 (2.38–5.88)	3.83 (2.08–6.71)	0.934
TyG index	8.83 ± 0.61	9.03 ± 0.94	0.295
Hypertension (n, %)	18 (41.9%)	8 (36.4%)	0.791
Preoperation			
Baseline random GH (μg/L)	9.67 (5.21–27.70)	12.65 (8.33–40.00)	0.143
Baseline nadir GH (μg/L)	7.74 (4.63–22.60)	9.60 (6.16–40.00)	0.067
Baseline IGF-1 (ng/mL)	597.00 (445.00–772.00)	586.00 (515.25–681.25)	0.934
Baseline IGF-1 index	2.42 ± 0.75	2.33 ± 0.71	0.659
Baseline PRL (μg/L)	10.50 (7.12–18.90)	22.14 (12.36–91.90)	**0.001**
Hyperprolactinemia (n, %)	9 (20.9%)	12 (54.5%)	**0.011**
Hypopituitarism (n, %)	7 (16.3%)	7 (31.8%)	0.204
Postoperation			
Postoperative GH (μg/L)	2.49 (1.57–6.84)	7.30 (3.18–11.08)	**0.011**
Postoperative GH < 1μg/L (n, %)	6 (14.0%)	2 (9.1%)	0.706
Follow-up			
Follow-up duration (months)	12.00 (6.00–37.00)	12.00 (4.75–20.00)	0.128
Random GH at follow-up (μg/L)	1.32 (0.48–3.54)	3.55 (0.75–11.25)	0.052
IGF-1 at follow-up (ng/mL)	252.00 (198.00–387.00)	432.50 (275.00–750.25)	**0.013**
IGF-1 index at follow-up	1.31 ± 0.63	1.82 ± 0.94	**0.011**
Biochemical remission (n, %)	16 (37.2%)	4 (18.2%)	0.159
Second operation (n, %)	2 (4.7%)	2 (9.1%)	0.599

GH, growth hormone; PRL, prolactin; BMI, body mass index; HbA1c, glycated hemoglobin A1c; FPG, fasting plasma glucose; HOMA-β, homeostasis model assessment for β cell function; HOMA-IR, homeostasis model assessment for insulin resistance; TyG, triglyceride-glucose; IGF-1, insulin-like growth factor-1.

The bold values represent the P-values that are statistically significant.

Preoperative endocrine assessments revealed that the two groups had similar random GH levels, nadir GH levels, and IGF-1 index at baseline. As expected, hyperprolactinemia was more common in patients with GH/PRL positive tumors (54.5% vs. 20.9%, *P* = 0.011), and the median PRL level was significantly higher in the GH/PRL positive tumors than in the GH positive tumors [22.14 (12.36–91.90) vs. 10.50 (7.12–18.90), *P* = 0.001]. After surgery, the patients with GH/PRL positive tumors had significantly higher postoperative GH levels [7.30 (3.18–11.08) vs. 2.49 (1.57–6.84), *P* = 0.011] and IGF-1 index during follow-up (1.82 ± 0.94 vs. 1.31 ± 0.63, *P* = 0.011). Although there was no significant difference, patients with GH/PRL positive tumors tended to have a lower biochemical remission rate (18.2% vs. 37.2%, *P* = 0.159) than patients with GH positive tumors.

## Discussion

To our knowledge, this is the first study to systematically investigate the clinicopathological characteristics and treatment outcomes of acromegaly patients with PIT1/SF1 tumors. Additionally, PIT1 tumors were further subdivided into GH positive and GH/PRL positive tumors on the basis of the expression of GH and PRL by IHC, and the clinical and prognostic characteristics of the groups were compared. The results indicated that 1) PIT1/SF1 tumors, as a specific subtype of plurihormonal tumors, were not rare and represented approximately 30.0% of the acromegaly patients in this cohort. Furthermore, despite higher baseline IGF-1 levels, the demographic, imaging, metabolic, and prognostic features of patients with PIT1/SF1 tumors were similar to those of patients with PIT1 tumors in the context of acromegaly. 2) Compared with those with GH positive tumors, patients with GH/PRL positive tumors, characterized by their earlier age at diagnosis and male predominance, tended to exhibit a lower biochemical remission rate.

Acromegaly is a heterogeneous disease with complex pathological subtypes. The WHO classification categorizes tumors composed of a single cell population expressing multiple TFs as plurihormonal tumors (6). PIT1/SF1 tumors, the main subtype of plurihormonal tumors in acromegaly, were previously considered rare (8). Based on pangenomic analysis of 134 PitNETs, Neou et al. first reported that the gonadotroph marker NR5A1, which encodes SF1, was also expressed in GNAS wild-type somatotroph tumors (16). Rymuza et al. subsequently analyzed the RNA sequencing and genome-wide DNA methylation results of 48 somatotroph tumors and confirmed that there was a group of tumors co-expressing NR5A1 (17, 18). Recently, Dottermusch et al. summarized previous research and proposed the term “somatogonadotroph PitNET” for tumors with PIT1/SF1 co−expression (19).

The incidence of PIT1/SF1 tumors varies across studies and ranges from 0.5% to 8% in studies conducted on all types of pituitary adenomas with or without function (20, 21). Dottermusch et al. first reported that 52% of somatotroph tumors stained positive for PIT1 and SF1 (19). In our cohort, PIT1/SF1 tumors accounted for approximately 30.0% of acromegaly patients, which is lower than the prevalence reported in prior study. Geographic and ethnic disparities and the divergence of the study subjects may have contributed to this difference (22). The Dottermusch et al. study solely focused on somatotroph tumors and excluded MSTs and MSLTs, whereas in our study, we included all of the above. Therefore, strict inclusion criteria may inflate the prevalence of PIT1/SF1 tumors, and further studies are necessary to validate our findings.

To date, there have been few studies in which the clinicopathological features of acromegaly patients with PIT1/SF1 tumors were investigated. One study by a Chinese neurosurgical team revealed that PIT1/SF1 tumors exhibited more aggressive behavior and had a poorer prognosis (more postoperative complications but similar biochemical remission) than PIT1 tumors in a cohort of 215 PitNETs (21). Furthermore, the team retrospectively reviewed the clinical data of 193 patients with growth hormone-secreting pituitary adenoma (GHPA) and reported that the tumor sizes of GHPAs with multiple TF positivity were smaller and that these tumors were less invasiveness; however, the rate of hormonal remission was lower than that for GHPAs with only PIT1 positivity (23). In contrast to earlier findings, our results revealed that the clinical and prognostic features of patients with PIT1/SF1 tumors were similar to those of patients with PIT1 tumors. The discrepancy in the subjects significantly contributed to the differences between our study and other studies. In contrast to our study which focused on acromegaly, Wang et al. included nonfunctional adenomas, and the proportion of nonfunctional adenomas among PIT1/SF1 tumors was significantly higher than that among PIT1 tumors (40.4% vs. 18.2%, *P* = 0.003) (21). The study has shown that nonfunctional macroadenomas in surgically treated patients tended to have larger tumor diameters and be more invasive than GH macroadenomas (24). On the other hand, in the study by Zhang et al., GHPAs with multiple TF positivity included not only PIT1/SF1 tumors (63.5%) but also PIT1/TPIT tumors (28.8%) and PIT1/SF1/TPIT tumors (7.7%). As mentioned in the discussion, the expression of ACTH in GHPAs with multiple TF positivity may lead to a poorer prognosis (23). Furthermore, SF1 tumors frequently manifest as nonfunctional tumors with normal serum FSH and LH levels (6). Consequently, PIT1/SF1 tumors typically present with clinical manifestations related only to excess GH in acromegaly (25, 26). The above evidence also supports our findings.

For the first time, we found that acromegaly patients with PIT1/SF1 tumors had significantly higher baseline IGF-1 levels than patients with PIT1 tumors. We suspect that the gastric inhibitory polypeptide receptor (GIPR) plays a role in it. Upon binding GIP, GIPR activates the signaling pathway and ultimately promotes GH synthesis and secretion (27). Therefore, GIPR^+^ somatotroph adenomas had higher baseline serum IGF-1 levels than GIPR^-^ adenomas (28). Recent research has demonstrated that SF1 is closely correlated with GIPR (29). Consistent with prior research, Rymuza et al. reported that GIPR-high somatotroph tumors also highly expressed NR5A1 and concluded that activated GIPR related signaling pathways triggered the expression of NR5A1 (17). Collectively, the high expression of GIPR in PIT1/SF1 tumors may account for higher IGF-1 levels than in PIT1 tumors in acromegaly.

GH and PRL cosecreting tumors have emerged as a focus in recent years, but the precise definition of these tumors is still lacking in the current literature. Some previous studies classified somatotroph tumors with hyperprolactinemia as GH and PRL cosecreting tumors (10, 30), whereas other authors considered cosecreting tumors when tumors stained positive for both GH and PRL by IHC (31–33). In the latest multicenter retrospective study conducted on 604 acromegaly patients, the authors suggested that cosecreting tumors should be considered when the tumors stained positive for GH and PRL with elevated PRL levels or serum PRL levels above 100 ng/dL, regardless of the IHC result of PRL (34). The limitation of the above inclusion criteria, as noted by Wildemberg et al., is that some somatotroph tumors with intratumoral PRL expression in up to 5% of cells have elevated PRL levels due to the stalk effect, and some patients with GH/PRL positive tumors may not present with hyperprolactinemia (35). Given that the 2022 WHO classification recommends categorizing PitNETs on the basis of pituitary TFs combined with classical pituitary hormones and the cutoff of 10% for PRL positive cells favors the diagnosis of somatolactotroph tumors (36), we consider the PIT1 tumor to be a GH/PRL positive tumor when more than 10% of cells are positive for PRL by IHC.

This study is the first to evaluate the clinical and prognostic characteristics of GH/PRL positive tumors in the context of PIT1 positivity. In accordance with previous studies (10, 30, 34), we found that patients with GH/PRL positive tumors were younger at diagnosis than those with GH-positive tumors and predominantly male. Moreover, there is a debate regarding the tumor size and invasiveness of GH/PRL positive tumors. Some studies reported that these tumors were larger and more invasive (30, 34), while others did not find a difference (31, 32). In our series, no differences were found in tumor diameter or the prevalence of invasive tumors between GH/PRL positive and GH positive tumors. The possible reason for this discrepancy is the varying proportions of MSLTs among the subjects in different studies. Lv et al. demonstrated that MSLTs were aggressive tumors characterized by a larger tumor size, greater invasiveness, and worse biochemical remission (33). We hypothesize that the proportion of MSLTs is lower in our series.

Whether somatotroph tumors cosecreting PRL affect biochemical remission remains controversial (37). Araujo and Wang (10, 34) detected no difference in biochemical remission rates between GH/PRL positive and GH positive tumors. In contrast, a worse prognosis in patients with GH/PRL positive tumors has been reported in other studies (38). Our results revealed that patients with GH/PRL positive tumors had significantly higher IGF-1 levels during follow-up and tended to exhibit a lower biochemical remission rate than those with GH positive tumors, although the difference was not significant. Differences across studies probably resulted from the divergence of study subjects, remission criteria, and follow-up durations. Therefore, patients with GH and PRL stained tumors seem to require more time to achieve long-term biochemical remission (31), and we suggest that patients with these tumors require a close follow-up.

The current study has some limitations. As a single-center retrospective study, it was subject to the inherent limitations of retrospective analysis and possibly led to selection bias compared with multicenter research. Additionally, the relatively small sample size was a major limitation of our study. A considerable number of patients were excluded from the study because of insufficient medical records. Furthermore, the short follow-up duration also limits our ability to assess long-term prognosis. Multicenter prospective studies with long follow-up are needed to evaluate the clinical and prognostic characteristics of patients with different pathological types in acromegaly.

## Conclusion

PIT1/SF1 tumors, as a specific subtype of plurihormonal tumors, are not rare among acromegaly patients. Additional studies are necessary to clarify whether PIT1/SF1 tumors have distinctive clinical and prognostic features in acromegaly. Patients with GH/PRL positive tumors differ from those with GH positive tumors in terms of clinical manifestations and prognosis, and these patients may require more attention during long-term follow-up.

## Data Availability

The raw data supporting the conclusions of this article will be made available by the authors, without undue reservation.

## References

[1] Fleseriu M Langlois F Lim DST Varlamov EV Melmed S. Acromegaly: pathogenesis, diagnosis, and management. Lancet Diabetes Endocrinol. (2022) 10:804–26. doi: 10.1016/s2213-8587(22)00244-3 36209758

[2] Gadelha MR Kasuki L Lim DST Fleseriu M. Systemic complications of acromegaly and the impact of the current treatment landscape: an update. Endocr Rev. (2019) 40:268–332. doi: 10.1210/er.2018-00115 30184064

[3] Zendran I Gut G Kaluzny M Zawadzka K Bolanowski M. Acromegaly caused by ectopic growth hormone releasing hormone secretion: A review. Front Endocrinol (Lausanne). (2022) 13:867965. doi: 10.3389/fendo.2022.867965 35757397 PMC9218487

[4] Lopes MBS. The 2017 World Health Organization classification of tumors of the pituitary gland: a summary. Acta Neuropathol. (2017) 134:521–35. doi: 10.1007/s00401-017-1769-8 28821944

[5] Asa SL Casar-Borota O Chanson P Delgrange E Earls P Ezzat S. From pituitary adenoma to pituitary neuroendocrine tumor (PitNET): an International Pituitary Pathology Club proposal. Endocr Relat Cancer. (2017) 24:C5–8. doi: 10.1530/ERC-17-0004 28264912

[6] Asa SL Mete O Perry A Osamura RY. Overview of the 2022 WHO classification of pituitary tumors. Endocr Pathol. (2022) 33:6–26. doi: 10.1007/s12022-022-09703-7 35291028

[7] Asa SL Mete O Riddle ND Perry A. Multilineage pituitary neuroendocrine tumors (PitNETs) expressing PIT1 and SF1. Endocr Pathol. (2023) 34:273–8. doi: 10.1007/s12022-023-09777-x 37268858

[8] Tang H Wang X Bie Z Yang Z Wang B Li S. Pituitary adenomas with multiple cell lineage combinations: clinicopathological features and short-term prognosis. J Neurosurg. (2023) 139:810–21. doi: 10.3171/2022.12.JNS222118 36708537

[9] Fookeerah P Varikatt W Shingde M Dexter MAJ McLean M. Somatostatin receptor expression and clinical outcome of multilineage pituitary tumours expressing PIT1 and SF1. Endocr Connect. (2023) 12:e230328. doi: 10.1530/EC-23-0328 37851558 PMC10563595

[10] Wang M Mou C Jiang M Han L Fan S Huan C. The characteristics of acromegalic patients with hyperprolactinemia and the differences in patients with merely GH-secreting adenomas: clinical analysis of 279 cases. Eur J Endocrinol. (2012) 166:797–802. doi: 10.1530/eje-11-1119 22334636

[11] Katznelson L Laws ER Jr Melmed S Molitch ME Murad MH Utz A. Acromegaly: an endocrine society clinical practice guideline. J Clin Endocrinol Metab. (2014) 99:3933–51. doi: 10.1210/jc.2014-2700 25356808

[12] Alberti KG Zimmet PZ. Definition, diagnosis and classification of diabetes mellitus and its complications. Part 1: diagnosis and classification of diabetes mellitus provisional report of a WHO consultation. Diabetes Med. (1998) 15:539–53. doi: 10.1002/(sici)1096-9136(199807)15:7<539::Aid-dia668>3.0.Co;2-s 9686693

[13] Guerrero-Romero F Simental-Mendia LE Gonzalez-Ortiz M Martinez-Abundis E Ramos-Zavala MG Hernandez-Gonzalez SO. The product of triglycerides and glucose, a simple measure of insulin sensitivity. Comparison with the euglycemic-hyperinsulinemic clamp. J Clin Endocrinol Metab. (2010) 95:3347–51. doi: 10.1210/jc.2010-0288 20484475

[14] Matthews DR Hosker JP Rudenski AS Naylor BA Treacher DF Turner RC. Homeostasis model assessment: insulin resistance and beta-cell function from fasting plasma glucose and insulin concentrations in man. Diabetologia. (1985) 28:412–9. doi: 10.1007/bf00280883 3899825

[15] Villa C Vasiljevic A Jaffrain-Rea ML Ansorge O Asioli S Barresi V. A standardised diagnostic approach to pituitary neuroendocrine tumours (PitNETs): a European Pituitary Pathology Group (EPPG) proposal. Virchows Arch. (2019) 475:687–92. doi: 10.1007/s00428-019-02655-0 31578606

[16] Neou M Villa C Armignacco R Jouinot A Raffin-Sanson ML Septier A. Pangenomic classification of pituitary neuroendocrine tumors. Cancer Cell. (2020) 37:123–34 e5. doi: 10.1016/j.ccell.2019.11.002 31883967

[17] Rymuza J Kober P Rusetska N Mossakowska BJ Maksymowicz M Nyc A. Transcriptomic classification of pituitary neuroendocrine tumors causing acromegaly. Cells. (2022) 11:3846. doi: 10.3390/cells11233846 36497102 PMC9738119

[18] Kober P Rymuza J Baluszek S Maksymowicz M Nyc A Mossakowska BJ. DNA methylation pattern in somatotroph pituitary neuroendocrine tumors. Neuroendocrinology. (2024) 114:51–63. doi: 10.1159/000533692 37699356

[19] Dottermusch M Ryba A Ricklefs FL Flitsch J Schmid S Glatzel M. Pituitary neuroendocrine tumors with PIT1/SF1 co-expression show distinct clinicopathological and molecular features. Acta Neuropathol. (2024) 147:16. doi: 10.1007/s00401-024-02686-1 38228887 PMC10791732

[20] Mete O Cintosun A Pressman I Asa SL. Epidemiology and biomarker profile of pituitary adenohypophysial tumors. Modern Pathol. (2018) 31:900–9. doi: 10.1038/s41379-018-0016-8 29434339

[21] Wang X Tang H Bie Z Wang Y Yuan R Zhang Z. Clinical and pathological features of pit1/SF1 multilineage pituitary neuroendocrine tumor. Neurosurgery. (2024) 95:94–102. doi: 10.1227/neu.0000000000002846 38289085

[22] Lavrentaki A Paluzzi A Wass JA Karavitaki N. Epidemiology of acromegaly: review of population studies. Pituitary. (2017) 20:4–9. doi: 10.1007/s11102-016-0754-x 27743174 PMC5334410

[23] Zhang Y Tang H Li S Bie Z Ma X Wu H. Co-expression of multiple transcription factors is associated with clinical features and endocrine prognosis in growth hormone-secreting pituitary adenomas. Endocrine. (2024) 87:788–99. doi: 10.1007/s12020-024-04082-x 39455511

[24] Zada G Lin N Laws ER Jr. Patterns of extrasellar extension in growth hormone-secreting and nonfunctional pituitary macroadenomas. Neurosurg Focus. (2010) 29:E4. doi: 10.3171/2010.7.FOCUS10155 20887129

[25] Mihajlovic M Pekic S Doknic M Stojanovic M Rasic D Miljic D. Plurihormonal pituitary neuroendocrine tumours - A single centre experience. Int J Surg Pathol. (2024) 32:470–7. doi: 10.1177/10668969231183712 37438981

[26] Dogukan FM Karatay H Yuzkan S Burhan S Erkan B Yilmaz-Ozguven B. Clinicopathological correlates of PIT1 and SF1-multilineage pituitary neuroendocrine tumors and the diagnostic utility of NKX2.2 immunohistochemistry in pituitary pathology. Arch Pathol Lab Med. (2024) 32:470–7. doi: 10.5858/arpa.2023-0543-OA 38649148

[27] Hage M Janot C Salenave S Chanson P Kamenický P. MANAGEMENT OF ENDOCRINE DISEASE: Etiology and outcome of acromegaly in patients with a paradoxical GH response to glucose. Eur J Endocrinol. (2021) 184:R261–r8. doi: 10.1530/eje-20-1448 33830942

[28] Hage M Chaligné R Viengchareun S Villa C Salenave S Bouligand J. Hypermethylator phenotype and ectopic GIP receptor in GNAS mutation-negative somatotropinomas. J Clin Endocrinol Metab. (2019) 104:1777–87. doi: 10.1210/jc.2018-01504 30376114

[29] Bates HE Campbell JE Ussher JR Baggio LL Maida A Seino Y. Gipr is essential for adrenocortical steroidogenesis; however, corticosterone deficiency does not mediate the favorable metabolic phenotype of Gipr(-/-) mice. Diabetes. (2012) 61:40–8. doi: 10.2337/db11-1060 PMC3237652 22043004

[30] Guo X Zhang R Zhang D Wang Z Gao L Yao Y. Hyperprolactinemia and hypopituitarism in acromegaly and effect of pituitary surgery: long-term follow-up on 529 patients. Front Endocrinol (Lausanne). (2021) 12:807054. doi: 10.3389/fendo.2021.807054 35154007 PMC8825499

[31] Rick J Jahangiri A Flanigan PM Chandra A Kunwar S Blevins L. Growth hormone and prolactin-staining tumors causing acromegaly: a retrospective review of clinical presentations and surgical outcomes. J Neurosurg. (2019) 131:147–53. doi: 10.3171/2018.4.Jns18230 30215558

[32] Varlamov EV Wood MD Netto JP Thiessen J Kim J Lim DST. Cystic appearance on magnetic resonance imaging in bihormonal growth hormone and prolactin tumors in acromegaly. Pituitary. (2020) 23:672–80. doi: 10.1007/s11102-020-01075-7 32870441

[33] Lv L Jiang Y Yin S Hu Y Chen C Ma W. Mammosomatotroph and mixed somatotroph-lactotroph adenoma in acromegaly: a retrospective study with long-term follow-up. Endocrine. (2019) 66:310–8. doi: 10.1007/s12020-019-02029-1 31368083

[34] Araujo-Castro M Biagetti B Menéndez Torre E Novoa-Testa I Cordido F Pascual Corrales E. Differences between GH- and PRL-cosecreting and GH-secreting pituitary adenomas: a series of 604 cases. J Clin Endocrinol Metab. (2024) 109:e2178–e87. doi: 10.1210/clinem/dgae126 38436926

[35] Wildemberg LE Gadelha MR. GH and prolactin co-secreting adenomas: it is time for a definition. J Clin Endocrinol Metab. (2024) 110:e192–3. doi: 10.1210/clinem/dgae262 38625822

[36] Van Laethem D Michotte A Cools W Velkeniers B Unuane D Andreescu CE. Hyperprolactinemia in acromegaly is related to prolactin secretion by somatolactotroph tumours. Horm Metab Res. (2020) 52:647–53. doi: 10.1055/a-1207-1132 32757187

[37] Agrawal N Ioachimescu AG. Prognostic factors of biochemical remission after transsphenoidal surgery for acromegaly: a structured review. Pituitary. (2020) 23:582–94. doi: 10.1007/s11102-020-01063-x 32602066

[38] De Marinis L Zuppi P Valle D Mancini A Bianchi A Lauriola L. A retrospective hormonal and immunohistochemical evaluation of 47 acromegalic patients: prognostic value of preoperative plasma prolactin. Horm Metab Res. (2002) 34:137–43. doi: 10.1055/s-2002-23197 11972303

